# The influence of scaffolding on intrinsic motivation and autonomous adherence to a game-based, unsupervised home rehabilitation program for people with upper extremity hemiparesis due to stroke. A randomized controlled trial

**DOI:** 10.21203/rs.3.rs-4438077/v1

**Published:** 2024-06-07

**Authors:** Gerard Fluet, Qinyin Qiu, Amanda Gross, Holly Gorin, Jigna Patel, Alma Merians, Sergei Adamovich

**Affiliations:** Rutgers, The State University of New Jersey; Rutgers, The State University of New Jersey; Rutgers, The State University of New Jersey; Rutgers, The State University of New Jersey; Rutgers, The State University of New Jersey; Rutgers, The State University of New Jersey; New Jersey Institute of Technology

**Keywords:** serious games, rehabilitation, hand, arm, telerehabilitation, stroke

## Abstract

**Background:**

This parallel, randomized controlled trial examines intrinsic motivation, adherence and motor function improvement demonstrated by two groups of subjects that performed a twelve-week, home-based upper extremity rehabilitation program. Seventeen subjects played games presenting eight to twelve discrete levels of increasing difficulty. Sixteen subjects performed the same activities controlled by success algorithms that modify game difficulty incrementally.

**Methods:**

33 persons 20 to 80 years of age, at least six months post stroke with moderate to mild hemiparesis were randomized using a random number generator into the two groups. They were tested using the Action Research Arm Test, Upper Extremity Fugl Meyer Assessment, Stroke Impact Scale and Intrinsic Motivation Inventory pre and post training. Adherence was measured using timestamps generated by the system. Subjects had the Home Virtual Rehabilitation System [1]systems placed in their homes and were taught to perform rehabilitation games using it. Subjects were instructed to train twenty minutes per day but were allowed to train as much as they chose. Subjects trained for twelve weeks without appointments and received intermittent support from study staff. Group outcomes were compared using ANOVA. Correlations between subject demographics and adherence, as well as motor outcome, were evaluated using Pearson Correlation Coefficients. Classification and Regression Tree (CART) models were generated to predict responders using demographics and baseline measures.

**Results:**

There were 5 dropouts and no adverse events. The main effect of time was statistically significant for four of the five clinical outcome measures. There were no significant training group by time interactions. Measures of adherence did not differ between groups. 21 subjects from both groups, demonstrated clinically important improvements in UEFMA score of at least 4.25 points. Subjects with pre training UEFMA scores below 53.5 averaged a seven-point UEFMA increase. IMI scores were stable pre to post training.

**Conclusions:**

Scaffolding did not have a meaningful impact on adherence or motor function improvement. A sparsely supervised program of game-based treatment in the home was sufficient to elicit meaningful improvements in motor function and activities of daily living. Common factors considered barriers to the utilization of telerehabilitation did not impact adherence or motor outcome.

**Trial registration:**

Clinical Trials.gov - NCT03985761, Registered June 14, 2019.

## Introduction

Despite decades of research attempting to remediate upper extremity impairments following stroke, a rehabilitation approach that elicits substantial improvements in function that do not decay over time has not been developed [2]. This points to a need for opportunities for persons with residual impairments following stroke to work on their arm and hand function away from the clinical environment with relative independence [3]. The use of traditional and technology-supported home-based rehabilitation programs has increased steadily in the last two decades and was further accelerated by the COVID - 19 pandemic [4]. Short term and directly supervised telerehabilitation programs produce comparable outcomes to clinic-based treatments [5, 6]. Longer programs and sparsely supervised programs have not been studied as well, and outcomes are less consistent. In general, adherence to programs of activity designed to improve or maintain motor function following a stroke is relatively low [7]. Multiple barriers to consistent performance of motor function training activities exist, including low motivation as well as a lack of interest in, or enjoyment of, training activities [8]. Multiple authors have proposed that game-based rehabilitation activities may help overcome these barriers and provide a solution to low adherence to home based rehabilitation programs [9–11]. This said, the published evidence presents a range of adherence rates to gamified, home based rehabilitation, suggesting that simply presenting a rehabilitation activity as a game might not result in across the board improvements in adherence [9, 12–17]. Multiple factors have been identified as possible causes for varied adherence to technology supported rehabilitation interventions in the home [9, 18, 19]. Various authors have speculated that personal attributes such as computer literacy, age and level of education, as well as socioeconomic factors such as employment status and income, might have an impact on the ability of persons with rehabilitation needs to accept and utilize technology based rehabilitation effectively [20, 21]. However, few studies have evaluated these speculations. This study will evaluate the impact of personal and socioeconomic factors on 1) adherence to a technology supported rehabilitation program and 2) the ability to make motor function improvements after participating in a technology supported rehabilitation program.

The gaming industry utilizes a wide variety of gaming mechanics, processes that govern the way a game flows, information is presented, and player success or failure is communicated to influence the frequency players pick up a game and play it, as well as the amount of time they play a game after initiating [22]. This study focused on scaffolding, a very common gaming mechanism that presents a relatively easy version of a game, followed by gradually ascending levels of difficulty as a participant succeeds [23]. This affords the participant immediate initial feelings of self-efficacy and then proceeds to challenge them. Appropriate levels of challenge [24] and feelings of self-efficacy [25] are both associated with higher levels of motivation, as is the clear knowledge of results feedback [24] a participant receives when they are presented with a new challenge after they succeed or they are required to repeat a level if they fail.

This study will utilize a parallel randomized clinical trial to examine the adherence levels of subjects with stroke performing a twelve-week, home-based upper extremity rehabilitation program incorporating simulations that used scaffolding to that of a control group of subjects that performed the same activities controlled by success algorithms that increase and decrease game difficulty incrementally and undetectably [26, 27]. We compared these approaches to controlling game difficulty using 1) the Intrinsic Motivation Inventory to measure the impact of the two approaches on motivation, 2) system-collected measurement of actual game play frequency and total training time to measure adherence and 3) clinical measures of upper extremity function to determine the effectiveness of the training programs. Our study focused on autonomous adherence to the training program by setting the subjects up with the system and having them perform their training without direct supervision or appointments in an attempt to approximate a sparsely supervised rehabilitation program conducted by a therapist.

## Methods

Subjects: Inclusion criteria were a) 20–80 years old, b) diagnosis of stroke confirmed from medical records, c) score greater than or equal to 22 on the Montreal Cognitive Assessment [28], d) visual field perception that allowed for attention to an entire 24” computer screen, e) proprioception sufficient to performing training activities without looking at their hand, f) Upper Extremity Fugl-Meyer Assessment (UEFMA) score of 10–60/66 [29] and g) receptive and expressive communication consistent with informed consent. Exclusion criteria were a) upper extremity orthopedic dysfunction that would limit upper extremity activity and b) chronic central nervous system pathology other than stroke. Subjects were recruited via local clinician referral and at stroke support groups. Subjects were screened and consented subjects by a study coordinator. After this they were assigned to one of either the Enhanced Motivation (EM) or Algorithm Controlled (AC) group using a random number generator (https://www.random.org/), following a simple randomization pattern. Subjects were blinded to treatment group allocation and the comparison being examined.

### Training System

The Home Virtual Rehabilitation System (HoVRS) is a computer based rehabilitation system designed to support independent training as well as remotely supervised training in the homes of persons with stroke (please see [1] for a detailed description of the system). HoVRS consists of two subsystems: 1) a patient-based system that presents rehabilitation games and 2) a cloud-based online data pipeline that allows for asynchronous monitoring and remote supervision. The patient-based system utilizes arm, wrist and hand position data collected by a Leap Motion Controller^™^ (LMC), an infrared camera-based tracking device. Images collected by the cameras are transmitted using the LMC’s tracking software, which transforms the images into three dimensional representations. The LMC’s application programming interface estimates relative wrist and finger positions, allowing the system to train specific motions of the fingers (flexion, extension and individuation) and wrist (flexion, extension, pronation, supination, radial and ulnar deviation). Tracking of hand position in 3d space allows for training of all elbow and shoulder movements as well. Upper extremity movements are used to control game play in a suite of games developed in the Unity 3D^™^ game engine. A variety of support systems, including mechanical arm supports and tabletop forearm platforms, were utilized as needed to maintain a participant’s hand in the active workspace of the LMC during arm, wrist or finger activities. Software consists of a library of twelve games, designed by our team to train shoulder/elbow, wrist and finger motions. Basic games train movements in isolation, while more advanced games train coordinated combinations of movements. Games are designed to accommodate a wide variety of active movement abilities via a calibration protocol that scales the amount of patient movement required to elicit avatar movement in the games. Game speeds, target / obstacle densities and sensory presentations are also scaled using the approaches described below to accommodate patients with moderate to severe impairments and challenge them as they progress.

### Treatment Programs

*Protocol* After randomization to one of the two interventions, subjects used the NJIT-HoVRS system to train movement of their shoulder, elbow, wrist, and fingers (Please see a detailed description of the HoVRS system in Qiu et al. 2021 [1]). Study teams consisting of a Physical Therapist and a technologist, who were not blinded to group allocation, set up the apparatus with all subjects in their homes at an initial visit and trained them to set up the system, open their assigned rehabilitation games, and play them.

*Treatment groups* The enhanced motivation (EM) group played two to five of the twelve available rehabilitation games, depending on their goals and the movements they wanted to train. These games provided the user with eight to twelve levels of gradually increasing difficulty and complexity (scaffolding). A screen announced each level change and the graphics for each new level changed substantially. Scoring opportunities increased at each new level as well. The algorithm control (AC) group also played two to five of the same twelve rehabilitation games. Game difficulty was modified using adaptive algorithms based on maintaining an eighty percent success rate over any given period of sixty seconds. Difficulty changes were designed to be incremental with the goal of making them imperceptible to subjects. Scoring opportunities and graphics did not change when the algorithms changed difficulty. Initially, subjects were assigned three simple simulations: one each for the shoulder / elbow, wrist, and fingers. Subjects were assigned games that targeted movements that limited their ability to perform daily functional tasks as determined by the study therapist during pre-testing. At follow up sessions, the study therapist updated the subjects’ training routines. Individual games were adjusted by increasing the amount of movement required to affect game play or increasing game speed, accuracy demands or target / obstacle densities. When simple games were mastered, games that combined wrist and hand movements (e.g. combining hand opening and pronation / supination) or games that combined finger movement with hand transport (e.g. moving the hand across a piano keyboard to press specific keys) were introduced. Subjects played the rehabilitation games in their homes independently, with on-line or in-person support as needed. All subjects were encouraged to play at least twenty minutes daily, but were allowed to play the games as much as they liked.

### Data Collection

#### All data were collected in subjects’ homes.

##### Demographic Data:

Demographic data, including subject age, occupation, employment status, level of education, a self-rating of computer literacy and the median income corresponding to each subject’s zip code, were collected prior to training.

##### Outcome Measures:

The impact of scaffolding on motivation was measured using the Intrinsic Motivation Inventory (IMI) [30]. Subjects completed a twelve-item version of the Intrinsic Motivation Interview (See Appendix 1) after the first and last training weeks to evaluate the impact of training game configuration on motivation to play the games, and the impact of extended play of the games (twelve weeks) on motivation as well as the correlation between levels of intrinsic motivation and adherence.

Adherence to the training programs was monitored and measured by tracking performance data collected by the system. Total treatment time over the twelve-week training period was estimated for each subject using computer timestamps of the files with performance data saved after each training session. In addition, the number of training sessions over the twelve-week training period was evaluated.

To measure the impact of training on changes in upper extremity motor function and examine the relationship between adherence to training on these changes, subjects completed the UEFMA [29], and Action Research Arm Test (ARAT) [31], just prior to and immediately after their participation in training. In addition, subjects completed the Hand, Activities of Daily Living, and Participation sub-scales of the Stroke Impact Scale (SIS) [32]. Tests were administered by a trained therapist blinded to group assignment.

### Data Analysis

#### Primary and secondary analyses

Anderson-Darling normality test was used to check for baseline data normality. Total treatment time, the primary analysis, was not normally distributed and thus analyzed using Mann–Whitney *U*tests for between group comparisons and Wilcoxon signed-ranks test for related samples. Secondary outcome measures were IMI, ARAT and SIS scores. A one-between, one-within repeated measures ANOVA was used to examine the effects of the treatment group (Enhanced Motivation, Algorithm Controlled) and testing time (Baseline, Post) on the secondary outcome measures.

#### Ancillary analyses

Classification and regression tree (CART) analysis, a machine learning procedure designed to create an optimal decision tree, was used to identify the optimal level of initial impairment for our intervention [33]. CART classification was used to evaluate the 1) ability of baseline clinical demographic factors to predict achieving a clinically important increase in UEFMA score (≥ 4.25 points as per Page [34]). Variables considered in the CART analysis were Training Group (EM or AC), baseline UEFMA score (BaseFM), Baseline ARAT score (BaseARAT), total training time (Minutes), total number of training sessions, (Sessions) median income for the subjects’ zip code (Income), baseline SIS hand subscale score (BaselineSIShand), baseline SIS activity of daily living subscale score (BaseSISADL), baseline SIS activity of participation subscale score (BaseSISPart), baseline IMI score (BaseIMI), age, months since CVA, and sex (M,F). All 28 subjects were used for the CART analysis. We tested the model using ten-fold cross validation. Performance of the models was evaluated using the area under the receiver operating characteristic curve (AUC).

Correlations between baseline demographics, clinical measures and training adherence were evaluated using Pearson Correlation Coefficients for continuous variables and Spearman Correlation Coefficients for categorical variables. All analyses were performed in Minitab 22.

## Results

### Subjects

**Figure 1:**
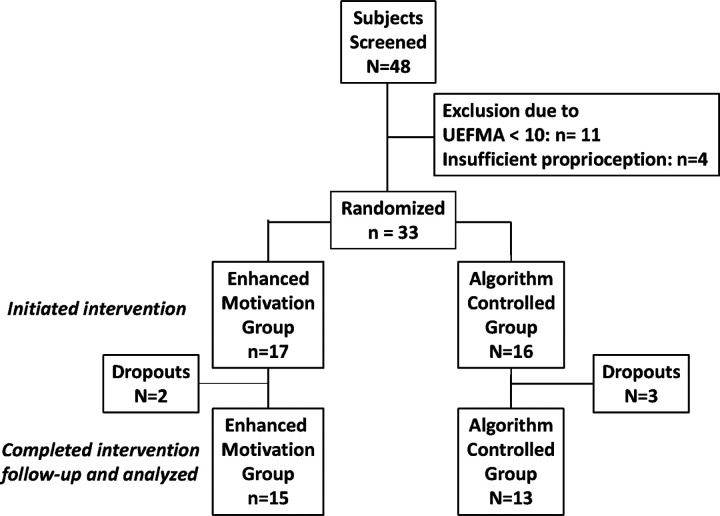
CONSORT Diagram

A total of 33 subjects (24 male and 9 female) satisfied the inclusion and exclusion criteria. Subject mean age was 57 (SD = 13). Mean time since stroke was 47 months (SD = 65) and baseline UEFMA was 43 (SD = 13). Subjects were randomized into EM (n = 17) and AC (n = 16) groups after baseline testing. There were five dropouts. There were no adverse events. For the remaining subjects, there were no statistically significant differences in baseline characteristics between EM and AC groups (Please see Fig. 1 and Table 1).

### Intrinsic Motivation Inventory

There were no statistically significant between group differences in IMI scores at baseline or post intervention testing, and there was no statistically significant training group by time interaction (See Table 2). The main effect of time was statistically significant (F (1,26) = 7.83, p = 0.007), and positive, suggesting that extended play of the rehabilitation games did not result in a decrease in intrinsic motivation. There were weak to moderate correlations between baseline as well as post intervention IMI and total training minutes, suggesting that there was a relationship between intrinsic motivation related to game play and adherence to the training protocol (See Table 3).

### Adherence

The EM group had two dropouts, and the AC group had three. One of the AC group dropouts did not enjoy the games. The other four dropouts reported difficulties with setup and playing the games as reasons for discontinuing training. There were no adverse events. Subjects that completed the protocol from both groups demonstrated substantial variance in adherence to the training protocol / total training time. EM group subjects’ training time ranged between 299 and 2672 minutes of training with a median training time of 966 (IQR = 442–1570) minutes. AC group subjects’ training time ranged between 165 and 1208 minutes of training with a median training time of 680 (IQR = 412–902) minutes. The within group variance and between group differences in the number of training sessions were smaller than those of total minutes. EM group subjects performed between 18 and 77 sessions. Mean number of sessions for the EM group was 48 (SD = 16). AC group subjects performed between 6 and 68 sessions. Mean number of sessions for the AC group was 37 (SD = 18) (See Table 2).

### Clinical Outcome Measures

(See Table 2) Main effect of time was statistically significant for UEFMA (F (1,26) = 112.4, p < 0.001), ARAT (F (1,26) = 29.1, p < 0.001), SIS-ADL (F (1,28) = 26.2, p < 0 .001), and SIS-Hand (F (1,26) = 5.7, p = 0.025). Subjects’ SIS - Participation scores did not change from pre to post-test. There were no statistically significant training group by time interactions for any of the clinical outcome measures. There were no statistically significant correlations between training time and any of the clinical outcome measures. 12 of the 15 subjects in the EM group and 9 of the 14 subjects in the AC group demonstrated improvements in UEFMA score that exceeded the published minimum clinically important difference (MCID) of 4.25 points for persons with chronic stroke [34]. 4 of the 15 subjects in the EM group and 7 of the 14 subjects in the AC group demonstrated improvements in ARAT score that exceeded the published minimum clinically important difference (MCID) for persons with chronic stroke [35].

### Ancillary analyses

**Figure 2:**
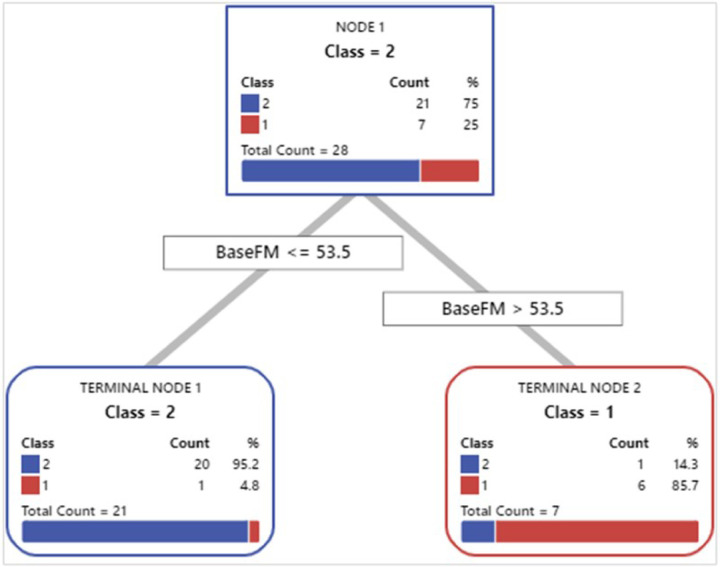
Optimal Tree produced by a 2 node CART classification analysis identifying responders (subjects demonstrating at least a 4.25-point increase in UEFMA score). Factors considered were: responders vs. age, months since stroke, baseline UEFMA score, baseline ARAT score, total training minutes, total training sessions, income, baseline SIS hand score, baseline SIS ADL score and baseline SIS participation score.

**Figure 3:**
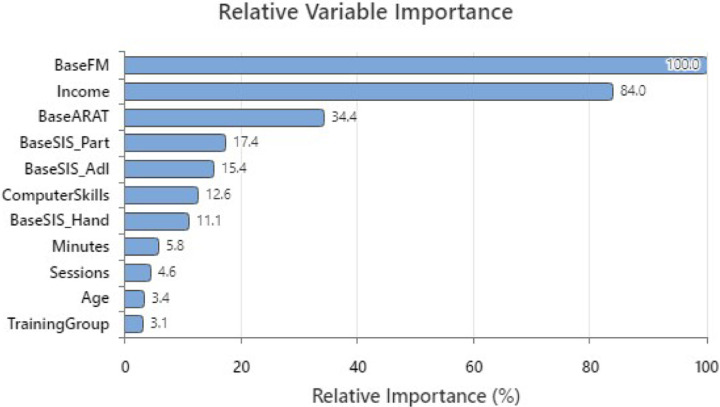
Receiver operating characteristic (ROC) curves. Training curve was generated using all 28 subjects that completed post testing. Testing curve was generated using ten-fold cross validation.

To identify subjects best able to benefit from our intervention, we used an exploratory predictive analytics approach using CART classification analysis, a decision tree algorithm that used changes in the UEFMA score from baseline to post-training to partition our subjects into two groups (see Fig. 2). Subjects who demonstrated a clinically important improvement greater than or equal to 4.25 points due to HoVRs training (21 subjects, Group1) had a BaselineFM scores of less or equal to 53.5, with a mean (SD) change in the UEFMA score of 7.0 (2.2). The second group (7 subjects, Group 2), defined by the CART algorithm, had initial BaselineFM scores larger than 53.5, with a mean (SD) change of 2.4 (1.49). Note that none of our subjects reached the maximal UEFMA score of 66. CART analysis performance was acceptable to excellent, with the area under the curve (AUC) for the training ROC of 0.905 and for the testing ROC of 0.738 (See Fig. 3). Odds ratio for training was 120. Odds ratio for testing was 15.

**Figure 4:**
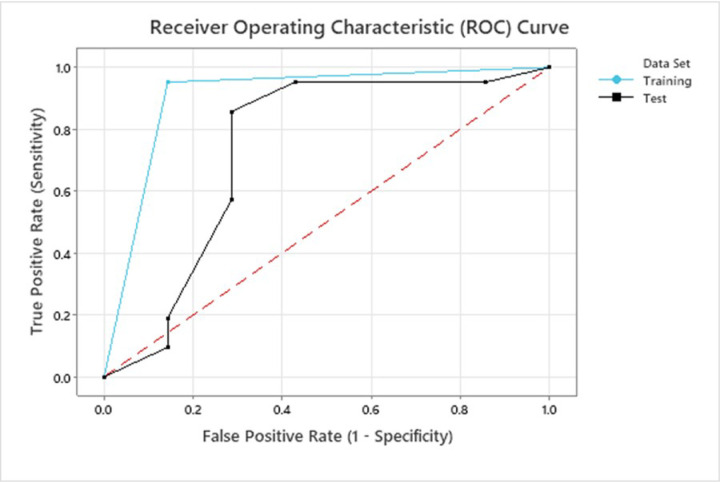
Relative value identification for each of the factors considered. Values below 100% describe the level of classification improvement that could be achieved if at least one node was split using this factor.

When considering the other factors included in this analysis (See Fig. 2), a single demographic factor, median income for the subjects’ zip code, was the next strongest predictor of clinically important improvement (See Fig. 4). Relative variable importance (RVI) of income demonstrated 84% of the predictive power of baseline UEFMA score. Subjects living in communities with lower median incomes were more likely to demonstrate clinically important differences. Computer skill level had substantially less predictive power (RVI = 12.6%). Four clinical baseline characteristics measures followed, Baseline ARAT score (RVI = 34.4%), Baseline SIS participation (RVI = 17.4%), Baseline SISADL (RVI = 15.4%) and Baseline SIS Hand (RVI = 11.1%). Subjects with lower baseline scores for these outcome measures were more likely to demonstrate clinically important differences. The two adherence measures, Baseline IMI and IMI Change, all other demographic measures and training group demonstrated trivial predictive power when compared to the baseline UEFMA score (See Fig. 4).

There were no statistically significant correlations between demographic factors and adherence. There were weak to moderate correlations between baseline as well as post intervention IMI and total training minutes, suggesting that there was a relationship between intrinsic motivation related to game play and adherence to the training protocol. (See Table 3) There were no statistically significant correlations between training time or number of training sessions and any clinical outcome measures (See Table 4).

## Discussion

This study examined the adherence of a group of persons with upper extremity hemiparesis due to stroke who performed one of two different game-based, autonomous training programs targeting their paretic arms, hands and fingers. The two programs differed in the level of explicit feedback related to success that they were provided during game play. The EM group, which was presented with more explicit feedback, demonstrated similar IMI scores immediately after the first week of training and immediately after the last week of training compared to the AC group that was provided less explicit feedback. Despite this similarity and the fact that there was a moderate correlation between IMI scores and total training time, the EM group demonstrated slightly larger median training times over the 12-week training program than the AC group. This suggests that there was some aspect of the interaction between the two training programs and subjects that differed, which was not captured by the IMI.

There were no statistically significant correlations between training time and improvements in clinical outcomes. The lack of a relationship between training time and outcome differs from some studies of the relationship between UE rehabilitation time and outcome [36] but is similar to other studies that cite a relatively weak relationship between training dosage and clinical outcomes after a minimum training threshold is achieved [37, 38]. This said, the number of subjects that demonstrated clinically important improvements in UEFMA and ARAT scores suggests that the training stimulus was strong enough even at lower training volumes to impact hand function.

Overall adherence to both training programs was modest. Dropout rates were 11% and 13% for the two groups and total training time was lower than many studies of home-based rehabilitation. It’s likely that this is due to the fact that 1) the intervention was relatively long (twelve weeks), and 2) subjects did not have to train by appointment. When comparing subjects in studies examining sparsely supervised, home based rehabilitation interventions, adherence rates and training time were better than those of subjects in a study by Standen [17] but not as good as those in a study by Rand [9].

The relatively high IMI scores and statistically significant increase in total IMI score over time might suggest that both of the training programs were relatively engaging over the course of training. The salience of training stimuli and engagement in training are both cited as factors influencing experience dependent neuroplasticity that underlies motor recovery post-stroke [39]. This might suggest that high levels of interest and engagement in training might be an important variable related to the consistent improvements in motor function in spite of relatively modest total training volumes.

Advanced age, lower levels of technology / computer literacy and lower levels of education have been cited as potential barriers to the use of and ability to benefit from technology supported rehabilitation approaches [40]. Interestingly, our data did not support these generalizations, with all three of these factors failing to make substantial contributions to the model predicting UEFMA improvement and the lack of correlation between adherence and these variables. We feel that this may be due to a general trend in increasing computer literacy / skill in older persons and/or a concerted effort to design the HoVRS system to be used by persons with minimal computer skills. Another design objective for this system was the ability to accommodate persons with relatively severe upper extremity motor impairments. We feel that the CART analysis, including subjects with baseline UEFMA scores below thirty in the cluster of subjects making more substantial improvements, suggests that this objective was achieved. With these statements made, it is obvious that further study designed to evaluate these assertions prospectively, in a larger group of subjects, is indicated before definitive conclusions can be made. Finally, our finding that higher levels of income predicted lower levels of UEFMA improvement using our system is ripe for further examination as well.

Limitations of this study include the lack of retention testing of clinical outcomes. Another limitation is the focus of this study on a single approach to motivation enhancement. This points to the need for continued study of this area of inquiry to investigate the additive effects of an expanded set of enhancement techniques that might include competition, cooperative play and narrative. Our subjects volunteered to participate in a study of technology supported rehabilitation which might limit our findings’ generalizability to persons who are highly averse to technology.

## Conclusions

This study examined the impact of scaffolding on adherence to a sparsely supervised home-based training program targeting the paretic upper extremity of persons with stroke. The effect of scaffolding elicited a non-significant difference in training time that had no effect on intrinsic motivation or improvements in motor function due to training. Across the board improvements in upper extremity motor function suggest that a sparsely supervised, game-based training program performed in the home can have meaningful, positive effects on arm, hand and finger function in persons with chronic hemiparesis due to stroke.

## Figures and Tables

**Table 1:** Baseline Demographic and Clinical Test Scores

	Algorithm Controlled n = 13	Enhanced Motivation n = 15	Baseline t-test	Study Mean n = 28
Age	55.87 (14.5)	58.00 (11.1)	0.674	56.86 (12.3)
Sex M/F	9/4	13/2		22/6
Months Since CVA	63.00 (84.0)	29.15 (28.8)	0.180	47.29 (64.8)
Median ZIP Income	95.00 (32.4)	102.31 (34.7)	0.577	98.39 (33.1)
Intrinsic Motivation Inventory	69.27 (6.8)	65.30 (6.0)	0.121	67.42 (6.6)
UEFMA	43.07 (12.0)	43.00 (14.3)	0.990	43.04 (13.0)
ARAT	32.33 (17.7)	26.08 (20.3)	0.401	29.43 (18.9)
Stroke Impact Scale - Hand	14.37 (5.7)	12.71 (4.8)	0.424	13.60 (5.3)
Stroke Impact Scale - ADL	37.08 (7.5)	35.10 (5.8)	0.453	36.16 (6.7)
Stroke Impact Scale - Participation	27.99 (6.7)	25.60 (8.4)	0.421	26.88 (7.6)

**Table 2:** Outcome Measure Scores (Standard Deviation), Δ = Change, **Median, [Interquartile Range]**

	IMI			Minutes	Sessions	UEFMA			ARAT			SIS Hand			SIS ADL	
	Pre	Post	Δ	Total	Total	Pre	Post	Δ	Pre	Post	Δ	Pre	Post	Δ	Pre	Post
EM	65.30 (6.0)	70.91 (6.8)	5.62 (4.4)	**966 [442–1570]**	37.15 (17.6)	43.00 (14.3)	48.23 (11.9)	5.23 (3.2)	26.08 (20.2)	31.69 (20.8)	5.62 (4.9)	12.71 (4.8)	14.46 (5.5)	1.81 (2.5)	35.10 (5.8)	38.49 (6.3)
AC	69.27 (6.8)	73.49 (6.1)	4.22 (5.7)	**680 [412–902]**	47.67 (16.4)	43.07 (12)	49.47 (11.3)	6.40 (2.5)	32.33 (17.7)	35.93 (17.5)	3.47 (3.9)	14.37 (5.7)	15.40 (5.5)	0.82 (3.7)	37.08 (7.4)	38.85 (6.6)
Group	67.42 (6.6)	72.29 (6.5)	4.87 (5)	**765 [440–1071]**	42.79 (16.4)	43.04 (12.9)	48.89 (11.4)	5.86 (2.8)	29.43 (18.9)	33.96 (18.9)	4.46 (4.5)	13.60 (5.3)	14.96 (5.4)	1.28 (3.28)	36.16 (6.7)	38.68 (6.4)

(Standard Deviation), Δ = Change, **Median, [Interquartile Range]**

**Table 3:** Correlations between demographics, and adherence measures

	Minutes	Sessions	UEFMAΔ	ARATΔ	SIS Hand Δ
Sessions	.769*				
UEFMAΔ	−.135	**−.099**			
ARATΔ	.163	**−.029**	**.287**		
SIS Hand Δ	.111	**.352**	**−.331**	**.178**	
SIS ADL Δ	−.328	**−.141**	**−.167**	**.033**	**.323**

* = p < .05, **bold = Spearman Correlation Coefficient**

**Table 4:** Correlations between demographics and adherence measures

	Minutes	Sessions	Age	Income	Computer Skills	Education	Baseline IMI
Sessions	.769*						
Age	.247	**.182**					
Income	.187	**.156**	**.358**				
Computer Skills	−.087	−.029	−.325	−.026			
Education	.028	−.175	.111	.245	.325		
Baseline IMI	.172	**.246**	**.107**	**.092**	−.195	−.310	
Post IMI	.459*	**.223**	**−.014**	**.067**	−.028	−.176	**0.703** *

* = p < .05, **bold = Spearman Correlation Coefficient**

## Data Availability

Data supporting this submission will be furnished upon written request to the corresponding author.

## Abbreviations

HoVRS: Home Virtual Rehabilitation System
CART: Correlation Coefficients. Classification and Regression Tree
UEFMA: Upper Extremity Fugl Meyer Assessment
IMI: Intrinsic Motivation Inventory
EM: Enhanced Motivation
AC: Algorithm Controlled
LMC: Leap Motion Controller
NJIT: New Jersey Institute of Technology
ARAT: Action Research Arm Test
SIS: Stroke Impact Scale
BBT: Box and Blocks Test
IQR: Interquartile Range
MCID: Minimum Clinically Important Difference
